# A pair of orthologs of a leucine-rich repeat receptor kinase-like disease resistance gene family regulates rice response to raised temperature

**DOI:** 10.1186/1471-2229-11-160

**Published:** 2011-11-15

**Authors:** Haitao Zhang, Yinglong Cao, Jing Zhao, Xianghua Li, Jinghua Xiao, Shiping Wang

**Affiliations:** 1 National Key Laboratory of Crop Genetic Improvement, National Center of Plant Gene Research (Wuhan), Huazhong Agricultural University, Wuhan 430070, China

## Abstract

**Background:**

Rice *Xa3/Xa26 *disease-resistance gene encodes a leucine-rich repeat (LRR) receptor kinase-type protein against *Xanthomonas oryzae *pv. *oryzae *(*Xoo*) and belongs to a multigene family. However, the functions of most genes in this family are unknown.

**Results:**

Here we report that two orthologs of this family, the *NRKe *from rice variety Nipponbare and *9RKe *from variety 93-11 at the *RKe *locus, have similar functions although they encode different proteins. This pair of orthologs could not mediate resistance to *Xoo*, but they were transcriptionally induced by raised temperature. Transcriptional activation of *NRKe *or *9RKe *resulted in the formation of temperature-sensitive lesion mimics, which were spots of dead cells associated with accumulation of superoxides, in different organs of the transgenic plants. These plants were more sensitive to high temperature shock than wild-type controls. Transgenic plants carrying a chimeric protein consisting of the LRR domain of NRKe and the kinase domain of Xa3/Xa26 developed the same lesion mimics as the *NRKe*-transgenic plants, whereas transgenic plants carrying another chimeric protein consisting of the LRR domain of Xa3/Xa26 and the kinase domain of NRKe were free of lesion mimic. All the transgenic plants carrying a chimeric protein were susceptible to *Xoo*.

**Conclusion:**

These results suggest that the *RKe *locus is involved in rice response to raised temperature. The LRR domain of RKe protein appears to be important to sense increased temperature. The RKe-involved temperature-related pathway and Xa3/Xa26-mediated disease-resistance pathway may partially overlap.

## Background

Disease resistance (*R*) genes that mediate race-specific resistance are important for plants in defending themselves from various pathogen attacks. Currently, many *R *genes have been characterized and most of them encode proteins sharing common features. The most prevalent domain of characterized R proteins is the leucine-rich repeat (LRR), which is the major determinant of pathogen recognition [1]. A relatively large number of LRR-containing R proteins belong to the nucleotide-binding site-LRR class [2]; most of the characterized *R *genes against *Magnaporthe oryzae*, which causes rice fungal blast, belong to this class [3]. The second class is the LRR receptor-like R proteins that consist of an extracellular LRR domain and a transmembrane motif [4]; no rice *R *gene of this class has been characterized so far. The last class includes the LRR receptor kinase-like R proteins, which are only identified in rice so far [5,6]. Both LRR and kinase domain are common structures in many signaling pathways. Plants have a large number of LRR receptor kinase-like proteins [7]. All the LRR receptor kinase-like proteins contain a transmembrane motif, which departs the LRR domain to stay outside of the plasma [7]. Thus, as cell surface receptors, the LRR receptor kinase-like proteins are thought to recognize special extracellular ligands via the LRR domain and initiate the downstream signaling through the intracellular kinase domain.

Rice *R *gene *Xa3/Xa26 *confers race-specific resistance to *Xanthomonas oryzae *pv. *oryzae *(*Xoo*) that causes bacterial blight, one of the most devastating diseases of rice worldwide and it encodes a LRR receptor kinase-like protein [6]. *Xa3/Xa26 *belongs to a tandem clustered multiple gene family in the long arm of rice chromosome 11 [8,9]. Point mutations with positive selection were a major force of the evolution of this family [9]. Both paralogs (in the same rice variety) and orthologs (in different rice varieties) of this family have similar tissue-specific expression patterns, suggesting that they may have similar functions [10,11]. In addition, at least some paralogs of this family have dosage effects, in which their disease resistance functions are associated with their transcript amounts [12,13]. The expression of *Xa3/Xa26 *is developmentally regulated. It has a lower level of expression at the early developmental stage, but a higher level of expression at the late developmental stage, which results in rice plants carrying *Xa3/Xa26 *being susceptible to some *Xoo *strains at the seedling stage but showing enhanced resistance to the same *Xoo *strains at the adult stages [12]. Rice plants constitutively overexpressing *Xa3/Xa26 *have an enlarged resistance spectrum, an increased level of resistance, and a whole growth-stage resistance without influencing their morphologies and agronomic performance [12,14]. In addition, the ortholog alleles at *Xa3/Xa26 *locus confer a durable resistance to *Xoo *[15]. Another paralog of the *Xa3/Xa26 *family, the *MRKa*, could not mediate resistance to *Xoo *when regulated by its native promoter, but *MRKa *could confer partial resistance to *Xoo *when regulated by a strong constitutive promoter [13].

To ascertain the functions of other paralogs of the *Xa3/Xa26 *family, we constitutively expressed two orthologs of this family, *NRKe *and *9RKe*, from two rice varieties, at the *RKe *locus. This pair of orthologs cannot mediate resistance to *Xoo *as do their paralogs *Xa3/Xa26 *and *MRKa *in the present experimental condition. However, they are involved in rice response to raised temperature.

## Methods

### Plasmid construction and rice transformation

To overexpress *9RKe *and *NRKe *in rice, these two genes were amplified using polymerase chain reaction (PCR) primers NRKe-F and NRKe-R (Additional file 1, Table S1) and genomic DNA from indica rice variety 93-11 (*Oryza sativa *L. ssp. *indica*) and bacterial artificial clone (BAC) OSJNBa0004O15 from japonica variety Nipponbare (*O. sativa *L. ssp. *japonica*) as template, respectively. The PCR products were digested with *Bam*HI and ligated into the transformation vector pU1301, which contained a maize ubiquitin gene promoter to drive the inserted gene [12].

To study the function of different domains of *NRKe*, two mutations of *NRKe *were generated. The kinase domain-deleted *NRKe*, the *NRKe-ΔK*, was created by PCR amplification using primers NRKe-F and B+E-3 and the LRR-transmembrane motif-deleted *NRKe*, the *NRKe-K*, was created by PCR amplification using primers RKe-6F and NRKe-R (Additional file 1, Table S1). Both truncated genes were amplified using Nipponbare BAC OSJNBa0004O15 as template. The PCR products then were ligated into pU1301 under the regulation of maize ubiquitin gene promoter. Two chimeric genes, *Be1 *and *Be2*, which consist of different fragments of *NRKe *and *Xa3/Xa26*, were amplified by overlap extension PCR [16]. PCR primers MKb-F, B+E-1, B+E-2, and NRKe-R were used for construction of *Be1 *and primers NRKe-F, B+E-3, B+E-4, and MKb-R were used for construction of *Be2 *(Additional file 1, Table S1). Both chimeric genes were amplified using BAC 3H8, which harbors *Xa3/Xa26*, from rice variety Minghui 63 (*O. sativa *L. ssp. *indica*) and Nipponbare BAC OSJNBa0004O15 as templates. The PCR products were ligated into pU1301 under the regulation of maize ubiquitin gene promoter.

All the constructs were respectively transferred into *Agrobacterium tumefaciens *strain EHA105 by electroporation. Rice transformation was performed by the *Agrobacterium*-mediated method using calli generated from mature embryos of rice variety Mudanjiang 8 (*O. sativa *L. ssp. *japonica*) [17].

### Pathogen inoculation

Plants were inoculated with Philippine *Xoo *strains PXO61, PXO86, PXO71, and PXO99 by the leaf-clipping method at the booting (panicle development) stage [18]. Disease was scored by measuring the percentage disease area (lesion length/leaf length) at 2 weeks after inoculation.

### Temperature treatment

Rice plants grown in a greenhouse at approximately 24°C were transferred to growth chambers for temperature experiments at the tillering stage. The growth chamber conditions were controlled as 70% humidity, 14-h light, and 10-h dark at either 24°C or 35°C. For heat shock, transgenic and wild-type plants were grown in the same pots and transferred to a 42°C growth chamber for 32 h (until almost all the leaves of one group in the pot became completely rolled and some leaves died). The plants were then recovered by maintaining them at room temperature for 3 d and phenotypes were recorded.

### Gene expression analyses

RNA gel blot analysis was performed as described previously [19]. A 343-bp probe for both *NRKe *and *9RKe*, digested using *Bam*HI and *Sac*I from the PCR product amplified using primers NRKe-F and NRKe-R (Additional file 1, Table S1), was used for hybridization. Quantitative reverse transcription-PCR (qRT-PCR) was performed using gene-specific primers (Additional file 1, Table S1), as described previously [20]. The expression of actin gene was first used to standardize the RNA sample for each qRT-PCR. For each gene, qRT-PCR assays were repeated at least twice, with each repetition having three replicates. Only the results in one repetition were presented when similar results were obtained in repeated experiments. The level of expression relative to control was presented.

### Promoter sequence analysis

The putative *cis*-acting elements in the promoter regions of *NRKe *and *9RKe *were predicted at PlantCARE http://bioinformatics.psb.ugent.be/webtools/plantcare/html/ and PLACE http://www.dna.affrc.go.jp/PLACE/ databases.

## Results

### Overexpression of *RKe *resulted in the formation of lesion mimics in rice plants

**Figure 1:**
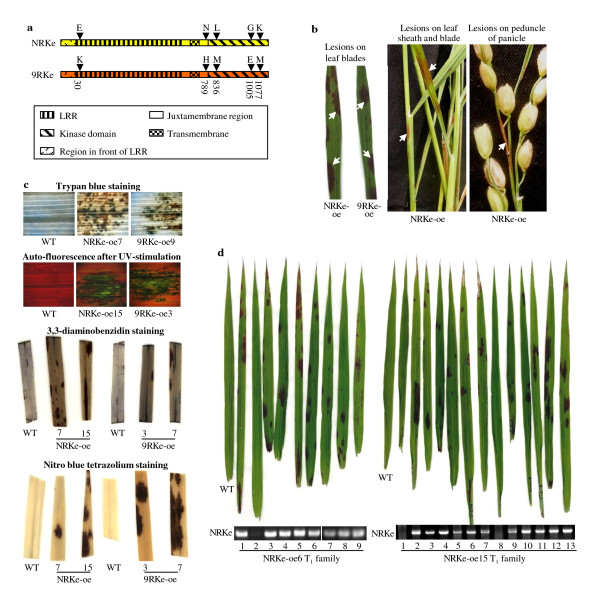
***NRKe *and *9RKe *caused the plants to form lesion mimics when overexpressed**. WT, wild-type Mudanjiang 8. (a) Comparison of deduced amino sequences encoded by *9RKe *and *NRKe*. Dark arrows show the polymorphic residues. Numbers indicate the polymorphic sites. LRR, leucine-rich repeat. (b) T_0 _transgenic plants (*NRKe*-oe and *9RKe*-oe) overexpressing *NRKe *or *9RKe *developed brown lesion mimics (white arrows) in different tissues. (c) The leaf lesions of the T_0 _*NRKe*-oe or *9RKe*-oe plants were associated with cell death and accumulation of H_2_O_2 _and superoxides. (d) Formation of lesion mimics on the leaves cosegregated with the existence of *NRKe *in two independent T_1 _families.

The paralog *RKe *of the *R *gene *Xa3/Xa26 *family only exists in some rice varieties [9]. The *RKe *genes in rice varieties 93-11 and Nipponbare, named *9RKe *[GenBank accession number: JN176871] and *NRKe *[JN176870], respectively, had 99.6% sequence identity. Both genes putatively encode proteins consisting of 1097 amino acids but with a five-residue difference (Figure 1a).

At least some paralogs of the *Xa3/Xa26 *family have dosage effects in rice-bacterium interactions [12,13]. To ascertain whether *NRKe *and *9RKe *also functioned in rice disease resistance, we constitutively overexpressed them in susceptible rice variety Mudanjiang 8, in which *RKe *was not detected. Twenty-two and 17 independent transformants transformed with *NRKe *and *9RKe *and named *NRKe*-oe and *9RKe*-oe were obtained, respectively. Some of the T_0 _plants showed overexpression of *NRKe *or *9RKe *(Additional file 1, Figure S1). All the T_0 _plants were inoculated with *Xoo *strain PXO61 at the booting stage and all the plants were susceptible as the wild-type Mudanjiang 8. After all the T_0 _plants were cut at about 12 cm above ground level after seeds had been harvested, the regenerated plants from the stubs were inoculated with other *Xoo *strains PXO86, PXO71, or PXO99. The transgenic plants were susceptible to these *Xoo *strains as the wild-type plants. Two independent T_1 _families generated from two T_0 _plants that overexpressed *NRKe *were inoculated with *Xoo *strain PXO61 for further analysis, but both families showed no difference from wild-type Mudanjiang 8. These results suggest that *NRKe *and *9RKe *may not have roles in rice-*Xoo *interaction.

Interestingly, 11 of the 22 *NRKe*-oe T_0 _plants and 12 of the 17 *9RKe*-oe T_0 _plants developed similar brown lesion mimics (i.e., spots that resemble infection) on leaf blades, leaf sheaths, and peduncles of panicles at the adult stage (Figure 1b). The lesion mimics could appear at any growth stage as small ones and developed to large ones during plant growth, but were restricted to an area around the initiation point. The lesion also could be formed spontaneously when the transgenic plants were grown in a sterilized tube or flask (Additional file 1, Figure S2). These results suggest that the formation of lesions is not related to pathogen infection.

Staining the leaves of the transgenic plants with visible lesion mimics using trypan blue, an indicator of dead cells [21], showed dark blue-stained spots at the sites around lesion mimics; such dark blue spots were not observed in the leaves of wild-type plants (Figure 1c). Autofluorescence, an indicator of phenolic compounds produced during cell death of hypersensitive response [22], was also detected at the sites of lesion mimics in the leaves of transgenic plants under the microscope after ultraviolet stimulation; no autofluorescence was detected in the leaves of wild-type plants (Figure 1c). The leaves with visible lesion mimics were also stained with 3,3-diaminobenzidin and nitro blue tetrazolium, the indicators of H_2_O_2 _and superoxides, respectively [23]. The leaves of the transgenic plants showed markedly increased dark staining at the sites of lesion mimics as compared to the leaves of wild-type plants after both 3,3-diaminobenzidin and nitro blue tetrazolium staining, indicating the accumulation of H_2_O_2 _and superoxides in the transgenic plants (Figure 1c). These results suggest that the lesions of the *NRKe*-oe and *9RKe*-oe plants were related to cell death, which may be associated with the accumulation of superoxides including H_2_O_2_. Because the *NRKe*-oe and *9RKe*-oe plants developed similar lesion mimics, only the *NRKe*-oe plants were used for some of the further analyses.

To determine whether the development of lesion mimics was due to overexpressed *NRKe*, two T_1 _families (*NRKe*-oe6 and *NRKe*-oe15) derived from two of the T_0 _plants that developed lesion mimics were further examined for their phenotypes and the existence of *NRKe*-oe construct. The development of lesion mimics on the leaves cosegregated with the existence of *NRKe *in the two families (Figure 1d). These results suggest that overexpressing *NRKe *caused the formation of spontaneous lesion mimics in rice.

### Formation of lesion mimics in *NRKe*-oe plants was temperature sensitive

Lesion mimic formation is a complex physiologic reaction in plants and may be caused by many biotic or abiotic factors. Because the *NRKe*-oe plants appeared not to be involved in rice-*Xoo *interaction, the formation of lesion mimics in these plants may be influenced by other environmental factors. Actually, two batches of *NRKe*-oe plants were generated in our experiments. The first batch of transgenic plants was planted in the field in early summer and marked lesion mimics were observed on their leaves when the plants grew to the booting stage during the summer. The second batch of transgenic plants was generated about 50 d later and was planted in the field in late summer. When the second batch of plants grew to the booting stage in the autumn, no obvious lesion mimic was observed in these plants. But after the second batch of plants was transferred to a greenhouse that had a higher temperature than the field condition, marked lesion mimics were formed on their leaves. These observations led us to infer that temperature may influence the formation of lesion mimics.

**Figure 2:**
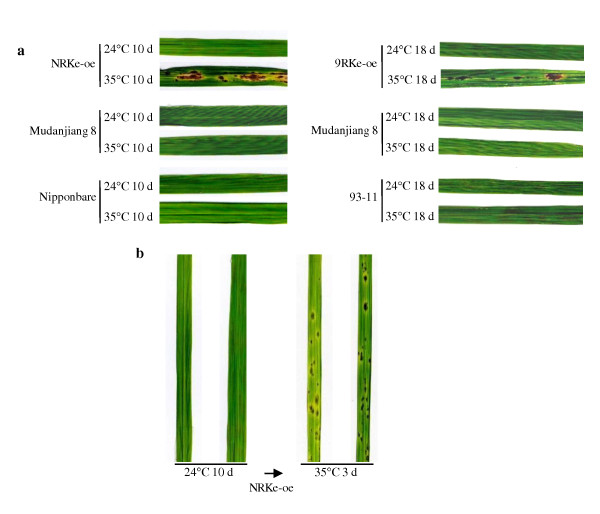
**Higher temperature induced formation of lesion mimics in T_1 _plants overexpressing *NRKe *(*NRKe*-oe15) and *9RKe *(*9RKe*-oe9)**. Mudanjiang 8 is the wild type. Nipponbare and 93-11 are the donors of *NRKe *and *9RKe*, respectively. (a) *NRKe*-oe and *9RKe*-oe plants developed large lesion mimics on their leaves after being grown at 35°C for 10 and 18 d, respectively. (b) The 24°C-pretreated *NRKe*-oe plants developed lesion mimics on their leaves at 3 d after transfer to 35°C conditions.

To test this inference, two identical sets of plants, including *NRKe*-oe and *9RKe*-oe plants, wild-type Mudanjiang 8, and rice varieties Nipponbare (the donor of *NRKe*) and 93-11 (the donor of *9RKe*) in each set, were grown in different temperatures at the tillering stage. The two sets of plants were first grown at 24°C and 35°C, respectively, for 10 to 18 d. All the plants grown at 24°C were free of lesion mimics. For the set of plants grown at 35°C, the *NRKe*-oe and *9RKe*-oe plants formed marked lesion mimics on their leaves at 10 and 18 d after being at 35°C, respectively, whereas the control Mudanjiang 8, Nipponbare, and 93-11 were free of lesions (Figure 2a). When the 24°C-pretreated *NRKe*-oe and control plants were transferred to 35°C, the transgenic plants but not control plants developed lesion mimics on their leaves even at 3 d after growing in 35°C (Figure 2b). These results suggest that higher temperature may induce the formation of lesion mimics in *NRKe*-oe and *9RKe*-oe plants.

### Temperature influenced the expression of *NRKe *and *9RKe*

**Figure 3:**
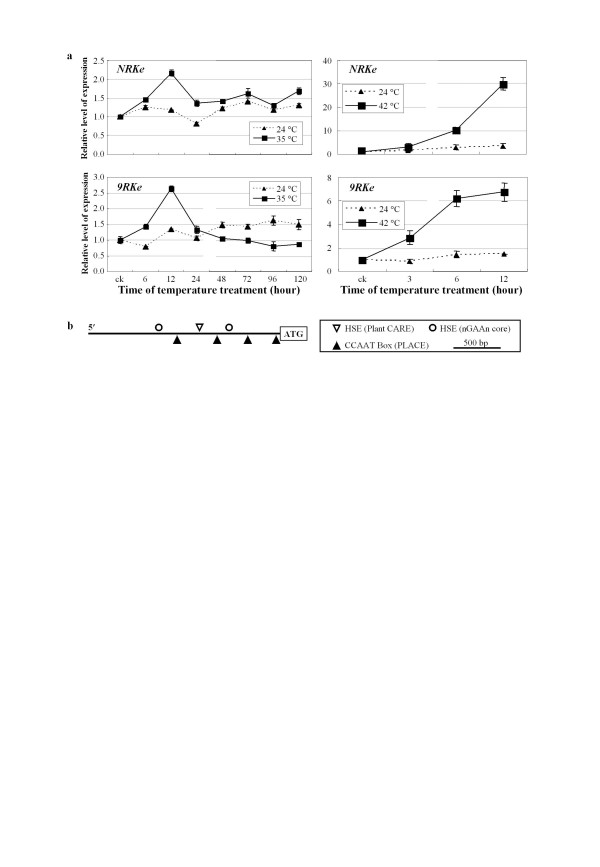
**Expression of *NRKe *and *9RKe *was induced after higher temperature treatment**. (a) Expression patterns of *NRKe *in rice variety Nipponbare and *9RKe *in rice variety 93-11 in different temperatures. ck, untreated control. (b) Putative heat-responsive *cis*-acting elements in the promoter regions of *NRKe *and *9RKe*. HSE (nGAAn core), heat shock element consisting of repeated core sequence of nGAAn in alternate orientation; HSE (PlantCARE), heat shock element found in PlantCARE database; CCAAT box (Place), CCAAT box found in PLACE database.

To ascertain whether temperature transcriptionally influenced *NRKe *and *9RKe*, we comparatively analyzed the expression of *NRKe *and *9RKe *in wild-type plants at different temperatures. At 35°C, the expression of *NRKe *and *9RKe *was markedly induced at 12 h after temperature treatment and then returned to the basal level of expression at 1 d after temperature treatment; the transcripts increased approximately 2- to 2.5-fold at 12 h after 35°C treatment compared to untreated control plants (Figure 3a). Raising the temperature to 42°C further increased the expression of *NRKe *and *9RKe*; the transcripts of *NRKe *and *9RKe *increased approximately 30- and 7-fold at 12 h after treatment, respectively. However, *NRKe *and *9RKe *expression was only slightly induced when the plants were kept at 24°C.

The promoter regions (approximately 2 kb upstream of translation start codon ATG) of *NRKe *and *9RKe *had 98% sequence identity. The two sequences were analyzed for putative *cis*-acting elements involved in heat stress response by searching different databases. The most canonical heat shock element (HSE) consists of a repeated core sequence of nGAAn ('n' indicating any nucleotide) in alternate orientation [24]. In the promoter regions of *NRKe *and *9RKe*, two imperfect nGAAn repeats were identified. The first nGAAn repeat was 'acTCaaTTCagGAAt' for *NKRe *and 'acTCaaTTCagGAta' for *9RKe*, and the second was 'aGAAtgGAgaacTCcatAAatcTCa' for both genes (Figure 3b; Additional file 1, Figure S3). The promoter regions of the two genes also harbored another putative HSE, which was complementary to the HSE 'AAAAAATTTC' of *Brassica oleracea *(Figure 3b; Additional file 1, Figure S3). Four CCAAT boxes were also identified in the promoter regions (Figure 3b; Additional file 1, Figure S3). The CCAAT box has been reported to act cooperatively with HSEs to increase promoter activity [25]. The occurrence of putative heat-responsive *cis*-elements and heat-induced expression suggest that *NRKe *and *9RKe *may function in early response to increased temperature.

### Activation of *NRKe *and *9RKe *expression influenced rice response to increased temperature

**Figure 4:**
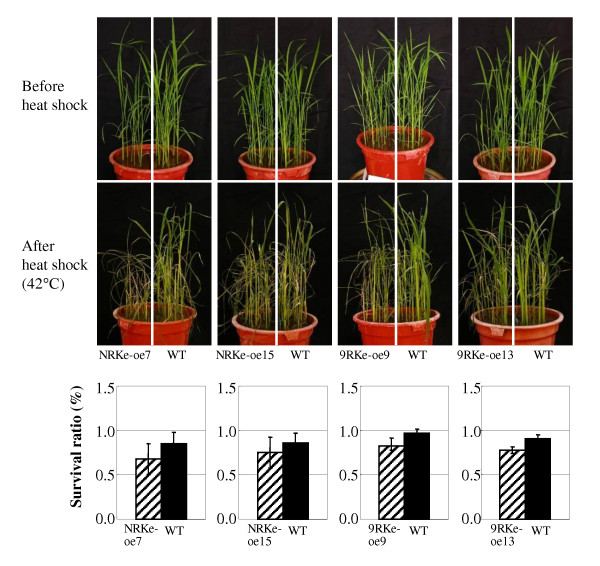
**Activation of *NRKe *or *9RKe *caused rice to be more sensitive to increased temperature**. *NRKe*-oe and *9RKe*-oe were T_2 _plants at 4- to 5-leaf stage. WT, wild-type Mudanjiang 8. Bars represent mean (2 technical replicates with each replicate contained 16-20 plants) ± standard deviation.

To examine the inference that *NRKe *and *9RKe *are involved in rice response to increased temperature, *NRKe*-oe or *9RKe*-oe plants and their wild-type plants were treated at 42°C. After treatment, the *NRKe*-oe and *9RKe*-oe plants appeared to be more sensitive to the heat shock than their corresponding wild-type plants (Figure 4). The transgenic plants showed lower survival rates compared to wild-type plants. These results further suggest that *RKe *orthologs influence rice response to temperature.

### The kinase domain of Xa3/Xa26 could replace the kinase domain of NRKe for promoting lesion mimics

**Figure 5:**
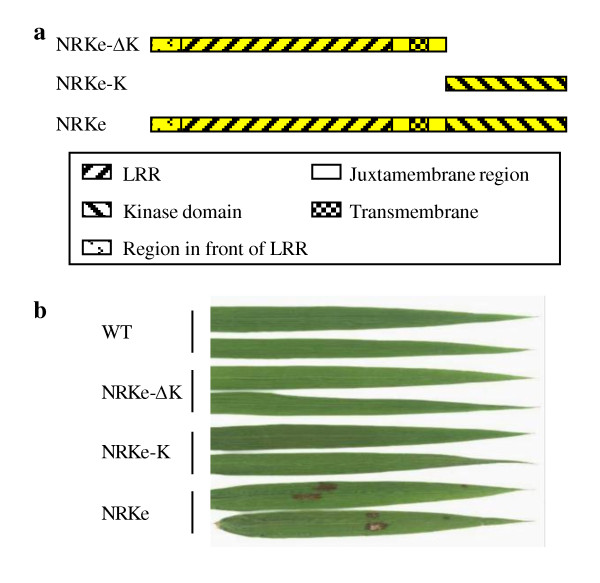
**Constitutive expression of truncated *NRKe*, the *NRKe-ΔK *and *NRKe-K *in rice**. (a) Predicted protein structures encoded by *NRKe*, *NRKe-ΔK*, and *NRKe-K*. (b) Leaves of transgenic plants overexpressing *NRKe-ΔK *(*NRKe-ΔK*-oe8, T_0 _generation), *NRKe-K *(*NRKe-K*-oe2, T_0 _generation), or *NRKe *(*NRKe*-oe15, T_1 _generation) at booting stage. WT, wild-type Mudanjiang 8.

*NRKe *putatively encodes a receptor kinase-type protein consisting of two domains, a LRR domain and a kinase domain, which are connected by a transmembrane motif. Usually, the two domains function together in a complicated way in signal transduction. One exception is rice XA21D, which is a truncated form of the LRR receptor kinase XA21 conferring resistance to *Xoo*. XA21D has only the LRR domain of XA21, but functions similarly as XA21 in rice-*Xoo *interactions [26]. To analyze the relationship of different domains of NRKe, two truncated *NRKe *fragments were, respectively, overexpressed in rice variety Mudanjiang 8. One truncated *NRKe *was *NRKe-ΔK*, which encoded protein did not have the kinase domain of NRKe; another truncated *NRKe *was *NRKe-K*, which only encoded the kinase domain of NRKe (Figure 5a). Eighteen and 18 independent transformants transformed with *NRKe-ΔK *and *NRKe-K*, and named *NRKe-ΔK*-oe and *NRKe-K*-oe, were obtained, respectively. In the same environment, *NRKe-*oe plants developed marked lesion mimics on their leaves, but *NRKe-ΔK*-oe and *NRKe-K*-oe plants were free of lesions (Figure 5b). These results suggest that a complete NRKe is important for the development of lesion mimics.

**Figure 6:**
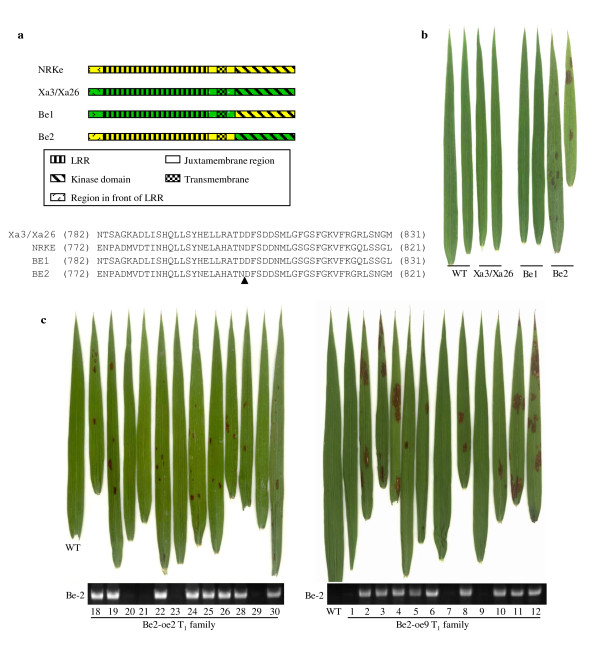
**The chimeric proteins and phenotypes of transgenic plants overexpressing chimeric gene**. (a) Schematic diagram of the proteins encoded by the chimeric genes *Be1 *and *Be2 *and the sequences around the fusion sites. Arrowhead indicates the fusion sites in Be1 and Be2 proteins. (b) Leaves of transgenic plants overexpressing *Xa3/Xa26 *(MKbFMDJ2 line)[12], *Be1 *(*Be1*-oe1, T_0 _generation), or *Be2 *(*Be2*-oe2, T_0 _generation) in the same genetic background. WT, wild-type Mudanjiang 8. (c) Formation of lesion mimics on the leaves cosegregated with the existence of *Be2 *in two T_1 _families at booting stage.

*NRKe *is the paralog of the *R *gene *Xa3/Xa26 *that confers race-specific resistance against *Xoo *in rice [6]. The predicted LRR and kinase domains of NRKe and Xa3/Xa26 proteins share 68% and 85% sequence identity, respectively (Additional file 1, Figure S4). To determine whether the LRR or kinase domain was critical for the development of lesion mimics, we constructed two chimeric genes, the *Be1 *and *Be2*, using the different fragments of *NRKe *and *Xa3/Xa26*, and constitutively overexpressed them in rice variety Mudanjiang 8. The *Be1 *consists of the sequences encoding the region in front of LRR, LRR, juxtamembrane region, and transmembrane region of Xa3/Xa26 and the sequence encoding the kinase domain of NRKe; the *Be2 *consists of the sequence encoding the region in front of LRR, LRR, juxtamembrane region, and transmembrane region of NRKe and the sequence encoding the kinase domain of Xa3/Xa26 (Figure 6a).

Eleven and 13 independent transformants transformed with *Be1 *and *Be2*, and named *Be1*-oe and *Be2*-oe, were obtained, respectively. All the *Be1*-oe plants were free of lesions as the wild-type control plants, but 8 of the 13 *Be2*-oe plants developed lesion mimics on their leaves at the booting stage (Figure 6b). To determine whether the development of lesion mimics was due to overexpressed *Be2*, two T_1 _families (*Be2*-oe2 and *Be2*-oe9) derived from two of the T_0 _plants that developed lesion mimics were further examined for their phenotypes and the existence of *Be2 *construct. The development of lesion mimics cosegregated with existence of *Be2 *in the two families (Figure 6c). These results suggest that the kinase domain of Xa3/Xa26 can replace the kinase domain of NRKe to induce formation of lesion mimics.

Because *Xa3/Xa26 *mediates rice resistance against *Xoo*, the T_0 _*Be1*-oe and *Be2*-oe plants were analyzed for their response to *Xoo*. Most of the T_0 _*Be1*-oe and *Be2*-oe plants were susceptible to *Xoo *strain PXO61 as the wild type. Two *Be1*-oe T_1 _families and two *Be2*-oe T_1 _families were further inoculated with PXO61. No increased resistance or susceptibility was associated with the existence of *Be1*-oe or *Be2*-oe construct in these T_1 _families (Additional file 1, Figure S5). These results suggest that the kinase domain of *NRKe *cannot replace the functions of the Xa3/Xa26 kinase domain in disease resistance.

## Discussion

The LRR receptor kinase-like plasma membrane proteins are the largest class of receptor-like kinases in plants and this class of proteins are well-known components in signal transduction during different physiologic activities [27]. For example, the CLAVATA1 regulates stem cell maintenance and differentiation in Arabidopsis [28]. BRI1 participates in the regulation of Arabidopsis growth and development via brassinosteroid signaling [29]. Rice Xa3/Xa26 and XA21 mediate race-specific and major disease resistance to *Xoo *[5,6]. The RPK1 functions in the abscisic acid signaling pathway related to germination, growth, and stomatal closure in Arabidopsis [30]. ERECTA specifies the size and shape of mature organs in Arabidopsis [31]. A LRR receptor kinase-like gene may be associated with the resistance to *Heterodera glycines *in soybean and the resistance to *H. glycines *is temperature sensitive [32,33]. The results presented here add another example that this type of protein, rice NRKe and 9RKe, appears to be involved in rice response to increased temperature. This inference is supported by the following evidences. First, both *NRKe *and *9RKe *were transcriptionally induced by higher temperature compared to control. Second, activation of this pair of orthologs resulted in rice more sensitive to high temperature.

### The LRR domain of RKe is critical to sense increased temperature

As receptor, the extracellular localized LRR domain of LRR receptor kinase-like protein functions to recognize and bind ligand, which includes proteins, peptides, or nonprotein components, in signal transduction [7]. For example, the LRR domain of BRI1 mediates brassinosteroid signaling by interacting with brassinolide, the active form of brassinosteroids [34]. Arabidopsis FLS2-mediated immunity is initiated by binding a bacterial flagellin-derived peptide Flg22 to its LRR domain [35]. Rice Xa21-regulated race-specific disease resistance is triggered by binding a sulfated peptide secreted by *Xoo *to its LRR domain [36]. Constitutive expression of the orthologs in the *RKe *locus resulted in the formation of lesion mimics, which was related to increased temperature (Figure 2). Thus, the appearance of lesion mimics in the *NRKe*- or *9RKe*-transgenic plants is a marker of rice response to raised temperature through *NRKe*- or *9RKe*-initiated signaling, although the appearance of lesion mimics did not represent a physiologic condition. The lesion mimic or lesion mimic-free phenotypes of different chimeric gene-carrying plants suggest that the LRR domain of the RKe protein is essential for the induction of lesion mimics (Figure 6). These results in turn indicate that formation of lesion mimics may be associated with the binding of temperature-associated ligand to the LRR domain.

Spontaneous formation of lesion mimics in the leaves of *NRKe*-, *9RKe*-, and *Be2*-transgenic plants cultured in sterilized containers was observed during tissue culture, which suggests that the ligand bound to the LRR domains of NRKe and 9RKe to induce the formation of temperature-sensitive lesion mimics was not from the changed environment. Thus, we argue that the ligand may be originated in rice. When the temperature rises, the ligand appears or its concentration is increased and RKe interacts with the ligand to trigger downstream reaction. However, further study is required to examine this hypothesis.

### The downstream pathways of RKe and Xa3/Xa26 may partially overlap

The intracellular localized kinase domain of LRR receptor kinase is the performer of transduction of the signal recognized by the LRR domain [7]. A previous report has revealed that a complete LRR receptor kinase-like protein including both the LRR and kinase domains in the Xa3/Xa26 family is important for conferring disease resistance; the kinase domain of a paralog in this family can partially replace the function of the kinase domain of resistance protein Xa3/Xa26 in response to *Xoo *[13]. Consistent with this previous report, our present results show that neither activation of the truncated NRKe lacking the kinase domain (NRKe-ΔK) nor activation of the kinase domain of NRKe (NRKe-K) can promote the formation of lesion mimics. The kinase domain of Xa3/Xa26 (in the case of Be2) can restore the function of NRKe-ΔK in promoting the formation of lesion mimics. However, the kinase domain of NRKe could not replace the kinase domain of Xa3/Xa26 (in the case of Be1) for *Xoo *resistance. These results suggest that the kinase domain of Xa3/Xa26 could afford all the functions of kinase domain of NRKe in formation of lesion mimics, but the latter could not provide the function of the former in disease resistance. Thus, some components of the downstream pathways of NRKe and Xa3/Xa26 may be shared.

The above inference is also supported by the fact that *NRKe *and *Xa3/Xa26 *belong to a tandem clustered *R *gene family; some amino acid sites of LRR domains encoded by the genes in this family are subject to positive selection, whereas the kinase domains encoded by these genes are evolutionarily conserved, suggesting the functional constraint of the kinase domains in this family [9]. However, not all the paralogs in this family possess the ability to confer disease resistance, although they have a similar tissue-specific expression pattern [11,13]. The present results suggest that some paralogs of the *R *gene family may be involved in other biological processes, like *NRKe *and *9RKe*.

## Conclusion

The tandem repeated paralogs of a haplotype and their orthologs in different haplotypes of *R *gene family provide different resistance specificities and a sequence reservoir for evolutionary forces to rapidly generate new *R *genes [37]. A previous report revealed that one paralog of tomato *R *gene *Pto *family, the *Fen*, confers sensitivity to fenthion, an organophosphorous insecticide [38]. Our results further suggest that the "defeated" *R *genes, like *NRKe *and *9RKe*, can also be involved in responses to raised temperature in rice, in addition to serving as the structural reservoir for creating new genes.

## Authors' contributions

HZ performed functional complementation and gene expression analyses and drafted the manuscript. YC and JZ provided biochemical and trans-genetic technical support. XL and JX provided molecular analysis support. SW contributed to data interpretation and to writing the manuscript. All authors read and approved the final manuscript.

## Supplementary Material

Additional file 1 **Supplemental table and figures**. **Table S1: ***PCR primers used for plasmid construction and gene expression analysis*. **Figure S1: ***Expression of NRKe and 9RKe in transgenic plants (T_0 _generation) analyzed by RNA gel blot. NRKe*-oe, NRKe-overexpressing plants; *9KRe*-oe, 9RKe-overexpressing plants; WT, wild-type Mudanjiang 8. **Figure S2: ***NRKe-overexpressing and Be2-overexpressing plants formed lesion mimics spontaneously in sterilized container during tissue culture*. **Figure S3: ***Alignment of promoter regions of NRKe and 9RKe*. The nucleotides immediately upstream of the translation start codon ATG are numbered as "-1". The putative heat-responsive *cis*-elements are underlined. HSE (nGAAn core), heat shock element consisting of repeated core nGAAn in alternate orientation; HSE (PlantCARE), heat shock element found in PlantCARE database; CCAAT box (Place), CCAAT box found in PLACE database. **Figure S4: ***Alignment of kinase domains of NRKe and Xa3/Xa26*. The solid black shade indicates different amino acid residues and the gray shade indicates residues with similarity. Asterisks (*) indicate conserved amino acid residues of protein kinase (Hanks SK et al. *Science *1998, **241**:42-52). The conserved subdomains are numbered and underlined according to Cao et al. (Cao Y et al. *Theor Appl Genet *2007, **115**:887-895). **Figure S5: ***Overexpression of Be1 or Be2 could not influence rice response to Xoo strain PXO61*. Positive transgenic plants were determined by PCR amplification of *Be1 *or *Be2 *using gene-specific primers (Additional file 1, Table S1). Wild type (WT) is Mudanjiang 8. Rb49 is a transgenic line carrying *Xa3/Xa26 *driven by its native promoter in Mudanjiang 8 background. Click here for file
