# Supplementary material for: A pair of orthologs of a leucine-rich repeat receptor kinase-like disease resistance gene family regulates rice response to raised temperature
Source: BMC Plant Biol. 2011 Nov 15;11:160. doi: 10.1186/1471-2229-11-160 (PMC3228767; doi:10.1186/1471-2229-11-160)
Supplement: Additional file 1 — Supplemental table and figures. Table S1: PCR primers used for plasmid construction and gene expression analysis. Figure S1: Expression of NRKe and 9RKe in transgenic plants (T0 generation) analyzed by RNA gel blot. NRKe-oe, NRKe-overexpressing plants; 9KRe-oe, 9RKe-overexpressing plants; WT, wild-type Mudanjiang 8. Figure S2: NRKe-overexpressing and Be2-overexpressing plants formed lesion mimics spontaneously in sterilized container during tissue culture. Figure S3: Alignment of promoter regions of NRKe and 9RKe. The nucleotides immediately upstream of the translation start codon ATG are numbered as "-1". The putative heat-responsive cis-elements are underlined. HSE (nGAAn core), heat shock element consisting of repeated core nGAAn in alternate orientation; HSE (PlantCARE), heat shock element found in PlantCARE database; CCAAT box (Place), CCAAT box found in PLACE database. Figure S4: Alignment of kinase domains of NRKe and Xa3/Xa26. The solid black shade indicates different amino acid residues and the gray shade indicates residues with similarity. Asterisks (*) indicate conserved amino acid residues of protein kinase (Hanks SK et al. Science 1998, 241:42-52). The conserved subdomains are numbered and underlined according to Cao et al. (Cao Y et al. Theor Appl Genet 2007, 115:887-895). Figure S5: Overexpression of Be1 or Be2 could not influence rice response to Xoo strain PXO61. Positive transgenic plants were determined by PCR amplification of Be1 or Be2 using gene-specific primers (Additional file 1, Table S1). Wild type (WT) is Mudanjiang 8. Rb49 is a transgenic line carrying Xa3/Xa26 driven by its native promoter in Mudanjiang 8 background. [file 1471-2229-11-160-S1.PDF]

A pair of orthologs of a leucine-rich repeat receptor kinase-like disease resistance gene family regulates rice response to raised temperature

Haitao Zhang, Yinglong Cao, Jing Zhao, Xianghua Li, Jinghua Xiao and Shiping Wang

Additional file 1: Supplemental table and figures

Table S1: *PCR primers used for plasmid construction and gene expression analysis*

Gene name (GenBank accession number)	Primer name	Forward primer (5'-3')	Reverse primer (5'-3')	Use
NRKe (JN176870) *9RKe* (JN176871)	NRKe-F/NRKeR	GCGGATCC[a]AAGCCAATCATCCCTTGAAATAG	GCGGATCC[a]GAAGCAAATATGGATGCAGAGG	Amplifying *9RKe*, *NRKe*, *NRKe-K*, *NRKe-ΔK*, *Be1*, and *Be2* for plasmid construction
NRKe	RKe-6F	AGCGGATCC[a]ATGGATTTCAGTGATGATAATATGTTGGG		Amplifying *NRKe-K* for plasmid construction
	B+E-2	ACCCCAACATATTATCATCACTGAAATCATCGGTTGCACGAAGAAG		Amplifying *Be1* for plasmid construction
	B+E-4	TGAGCTTGCTCATGCTACTAATGATTTCAGCGATGATAGCATGTTGG		Amplifying *Be2* for plasmid construction
NRKe *9RKe*	RKe-RF/RR	GAGCAGCGGTTTGGTGGTT	GTCGAGCCATTCGAAGAACAC	Quantitative RT-PCR
Xa3/Xa26	MKb-F/MK	TAGGATCC[a]ATGGCTCTTG	ATGGATCC[a]ACCACGAGAG	Amplifying *Be1* and *Be2* for

(DQ355952)	b-R	TTCGATTGCC	AGCGATGAAT	plasmid construction
	B+E-1	TTCAGTGATGATAATATGT TGGGGT		Amplifying *Be1* for plasmid construction
	B+E-3	ATCATTAGTAGCATGAGCA AGCTCA		Amplifying *Be2* and *NRKe*-Δ*K* for plasmid construction
Actin (X15865)	Actin-F/R	TGTATGCCAGTGGTCGTAC CA	CCAGCAAGGTCGAGACG AA	Quantitative RT-PCR

[a]The underlined nucleotides are the digestion site of *Bam*HI.

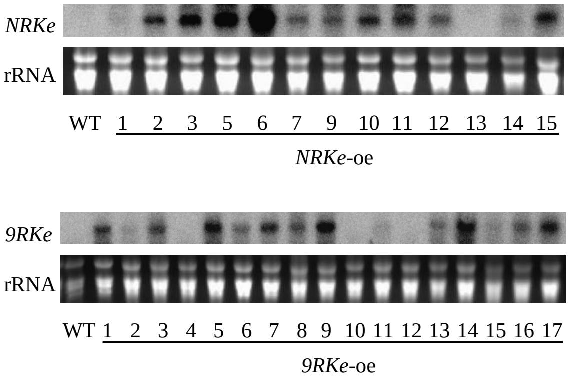

Figure S1: *Expression of NRKe and 9RKe in transgenic plants (T_0 generation) analyzed by RNA gel blot. NRKe*-oe, NRKe-overexpressing plants; *9KRe*-oe, 9RKe-overexpressing plants; WT, wild-type Mudanjiang 8.

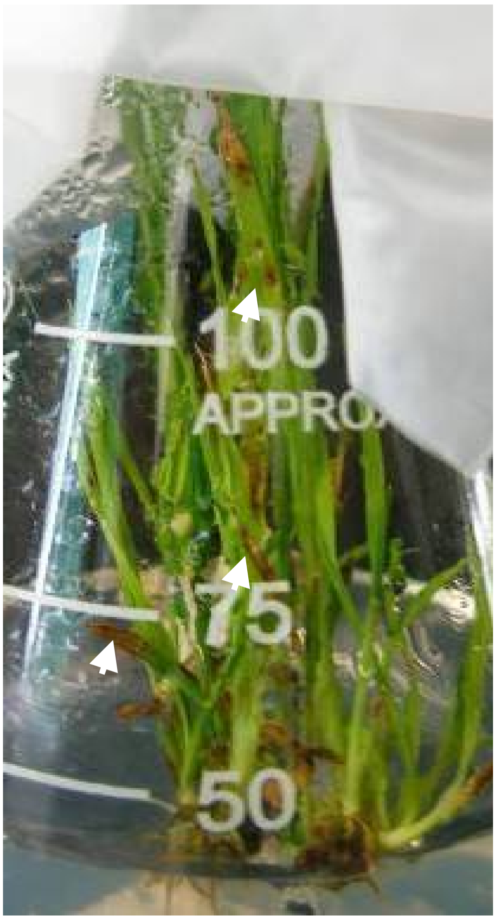

NRKe-oe plants

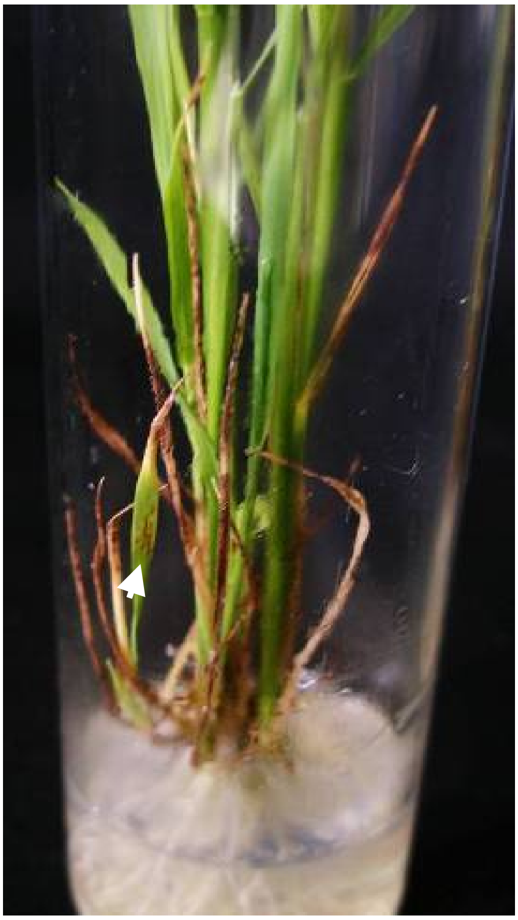

NRKe-oe plants

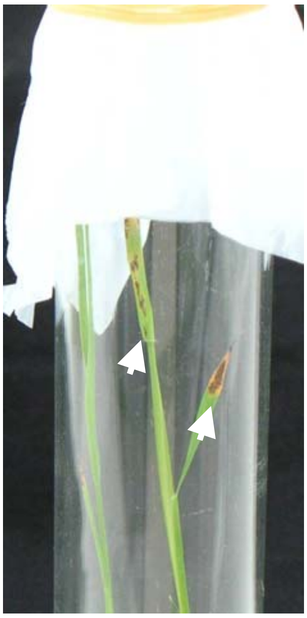

NRKe-oe plant

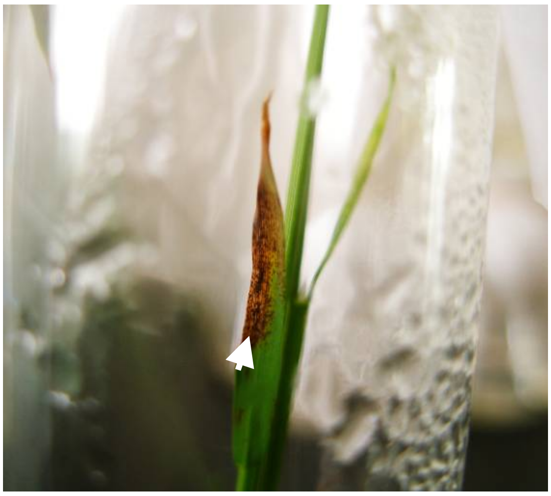

Be2-oe plant

Figure S2: *NRKe-overexpressing and Be2-overexpressing plants formed lesion mimics spontaneously in sterilized container during tissue culture.*

```
9RKe  GTGGCATATCCTATTAATTTCGTGCGATGGCTGGTAATAGGATGCCACGCCCTCGTCCTATGTGAAACAGCCACGTCATCTCGCGGGTGAATCCTTTCAT  -1933
NRKe  GTGGCATATCATATTAATTTCGTGCGATGGCTGGTAATAGGATGCCACGCCCTCGTCCTTTGTGAAACAGCCACGTCATCTCGCGGGTGAATCCTTTCAT  -1963

9RKe  TGATAGTGTGGTTTTGTGAAACTACACTACCAAAATATATGTACTTAAATGTCAAGTGTCAAAGTGTGTGGTTTTCTGCAACTTGGACCACAAAACGTAT  -1833
NRKe  TGATAGTGTGGTTTTGTGAAACTACACTACCAAAATATATGTACTTAAGTGTCAAGTGTCAAAGTGTGTGGTTTTCTGCAACTTGGACCACAAAACGTAT  -1863

9RKe  AGTTTTCTGAAATTTACTCTTTAAAAAATAACAGCCAAATAACACGGTATTCACTATTTAGTGCTTAAAAAAA-GAATCCAGAGTATTTCAATCACAAAA  -1733
NRKe  AGTTTTCTGAAATTTACTCTTTAAAAAATAACAGCCAAATAACATGGTATTCACTATTTAGTGCTTAAAAAAAAGAATCCAGAGTATTTCAGTCACAAAA  -1763

9RKe  ATTTCCGAAAAGTTTCCAACCGATTCTGAGTTCCAACGAAAATTGCCCTTATCATTTTTGATTCCGTTTCCGAGAAAATATTTCCGAATTCGTTTACGTT  -1633
NRKe  ATTTCCGAAAAGTTTCCGACCGATTCTGAGTTCCAGCGAAAATTGCCCTTATCATTTTTTATTCCGTTTCCGAGAAAATATTTCCGAATTCGTTTACGTT  -1663

9RKe  TCCGAAAAAATTACGACTGATAGATTCCGTTTTCGAAAATAAGTCTGGAATCCGGAAAGTTTCTATATCGTTTTCACCCCTATCTCACACGGATTAGGAT  -1533
NRKe  TCCGAAAAAATTACGACTGATAGATTCCGTTTTCGAAAATAAGTCTGGAATCCGGAAAGTTTCTATATCGTTTTCACCCCTATCTCACACGGATTAGGAT  -1563

9RKe  CCTCTGAAGTAAAATAAGAGGAACATGGTGGCTGACATGAGGGCCCCATCGCTTGCGGTTGCGGGCCCACCTATCAGTGATCACGTTAGTAGGGAGTTCA  -1433
NRKe  CCTCTGAAGTAAAATAAGAGGAACATGGTGGCTGACATGAGGGCCCCATCGCTTGCGGTTGCGGGCCCACCTATCAGTGATCACGTTAGTAGGGAGTTCA  -1463

9RKe  TATTAGAGGTTCTGCTTCCGTCTTACACTCACGCACAGGCGTGTGACCACCGTGATTGTGAAGATTGCAACCGCTGGCGACCGGCGGACGGCGGACGACA  -1333
NRKe  TATTAGAGGATCTGCTTCCGTCTTACACTCACGCACAGGCGTGTGACCACCGTGATTGTGAAGATTGCAACCGCTGGCGACCGGCGGACGGCGGGCGACA  -1363

9RKe  TATTTCTGTAACCGAAGGATGTAGTACTCAATTCAGGA-------TACATATAATCTGAACCTGAACATTACTGTCA-----------------------  -1263
NRKe  TATTTCTGTAACCGAAGGATGTAGTACTCAATTCAGGAATCAGGATACATATAATCTGAACCTGAACATTACTGTCAACACATGTCCATCTATACAGCCA  -1263
                  HSE (nGAAn core)
9RKe  GACAACGGAGATGCATACTTTTACCAGAATGCCTAGATTGACTAGTTATTCCCTTGAAAACAGTTTATAATTGATGGCGCCAAATTAAATGGTTGCATGC  -1163
NRKe  GACAACGGAGATGCATACTTTTACCAGAATGCCTAGATTGACTAGTTATTCCCTTGAAAACAGTTTATAATTGATGGCGCCAAATTAAATGGTTGCATGC  -1163

9RKe  AAGGCATAATTAATGGAAAGCTGAATAAATCTTAACCCAATCATCCCCTGATATAGTGACATTTTCTTGGTTGGTTGACCTTTTCACCTCTGGCTGCTGG  -1063
NRKe  AAGGCATAATTAATGGAAAGCTGAATAAATCTTAACCCAATCATCCCCTGATATAGTGACATTTTCTTGGTTGGTTGACCTTTTCACCTCTGGCTGCTGG  -1063
                                              CCAAT box
9RKe  TCGGCAGATTGACACTGCCCAAATTTATACTCCCTCATTCTCTAAATATTTGACGCCGTTAACTTTTTAAAACATATTTGACCGTTCGTCTTATTCAAAA  -963
NRKe  TCGGCAGATTGACACTGCCCAAATTTATACTCCCTCATTCTCTAAATATTTGACGCCGTTAACTTTTTAAAACATATTTGACCGTTCGTCTTATTCAAAA  -963

9RKe  ACTTTTACGAAATACGTAAAACTATATGTATACATAAAAGTACATTTAACAATGAATCAAATGGTAAGAAAAAAATTAATAATTGCTTAAATTTTTTGAA  -863
NRKe  ACTTTTACGAAATATGTAAAACTATATGTATACATAAAAGTACATTTAACAATGAATCAAATGGTAAGAAAAAAATTAATAATTGCTTAAATTTTTTAAA  -863
                                                                                 HSE (PlantCARE)
9RKe  TAAGACGAACGGTCAAACATGTTTAAAAAAATCAACGGTGTCAAACATTTAGGGATGGAGGGAGTACAATGTAGGCGAAAAAAAATATAATTGAAGGCAC  -763
NRKe  TAAGACGAACGGTCAAACATGTTTAAAAAAATCAACGGTGTCAAACATTTAGGGATGGAGGGAGTACAATGTAGGCGAAAAAAATTATAATTGAAGGCAC  -763

9RKe  CGGATGGATGCAATCAACAAAACAATGACACGTTCAGTAGATATAGAGAAAAACTGAATAATTCTCATGCCAATCATAAATAATGACATATTGGTTGACT  -663
NRKe  CGGATGGATGCAATCAACAAAACAATGACACGTTCAGTAGATATAGAGAAAAACTGAATAATTCTCATGCCAATCATAAATAATGACATATTGGTTGACT  -663
                                                                            CCAAT box
9RKe  TTTTGCCGGGTCCACCGATTGGCACTGCCCGAATGTATAGAACGATGGAGGGAAAACAGTTCATAAATGAAGGAGCCCAGATGGTTGCATCCACAAAGAA  -563
NRKe  TTTTGCCGGGTCCACCGATTGGCACTGCCCGAATGTATAGAACGATGGAGGGAAAACAGTTCATAATTGAAGGAGCCCAGATGGTTGCATCCACAAAGAA  -563

9RKe  TGGAGAACTCCATAAATCTCAGCGCTAATCATCCTCTGAAATAATGTCATGTTCTTGATTGACTTTTTCGCCGGCCGCGGATTGACACCCAAATTTATAG  -463
NRKe  TGGAGAACTCCATAAATCTCAGCGCTAATCATCCTCTGAAATAATGTCATGTTCTTGATTGACTTTTTCGCCGGCCGCGGATTGACACCCAAATTTATAG  -463
                            HSE (nGAAn core)
9RKe  AGCATATGGGAAGTGAAAACATTTTACAATTGAAGGCACCATACCATGTGGTTGCAAGGGCAGGGTAATGTTGAGCGCTGAATACATATCAAGCCAATCA  -363
NRKe  AGCATATGGGAAGTGAAAACATTTTACAATTGAAGGCACCATACCATGTGGTTGCAAGGGCAGGGTAATGTTGAGCGCTGAATACATATCAAGCCAATCA  -363
                                                                                         CCAAT box
9RKe  TCCGCTAAAATAATGCCATATTCTTGGTTGATTTCTTCACCGCACCAGACCAGCAGATTGATACCAACAAATTTATTGAGCAATGGAGGGAAAACAATTT  -263
NRKe  TCCGCTAAAATAATGCCATATTCTTGGTTGATTTCTTCACCGCACCAGACCAGCAGATTGATACCAACAAATTTATTGAGCAATGGAGGGAAAACAATTT  -263

9RKe  ATAATTGAAGGTGGCAGATGTTTACTTGAGCAGTGGAATAAATCTCAAGCTAATCATCCCCTGAAATTATGGCATCTTCTTGGTTGACTTCATCACCGAG  -163
NRKe  ATAATTGAAGGTGGCAGATGTTTACTTGAGCAGTGGAATAAATCTCAAGCTAATCATCCCCTGAAATTATGGCATCTTCTTGGTTGACTTCATCACCGAG  -163

9RKe  CAACCAGATTGACGCCGACAAGATTGATTAAGCAAGGTAGGGAAAAGAGTTTACAATTGAAGGCGATGACTGCATGCACAGCGGAATGATGTATTGGATA  -63
NRKe  CAACCAGATTGACGCCGACAAGATTGATTAAGCAAGGTAGGGAAAAGAGTTTACAATTGAAGGCGATGACTGCATGCACAGCGGAATGATGTATTGGATA  -63

9RKe  AATATAAGCCAATCATCCCTTGAAATAGCGCCATCTTGTTGGCAATGCATCAGAGCCCAAAC  -1
NRKe  AATATAAGCCAATCATCCCTTGAAATAGCGCCATCTTGTTGGCAATGCATCAGAGCCCAAAC  -1
                    CCAAT box (Place)
```

Figure S3: *Alignment of promoter regions of NRKe and 9RKe.* The nucleotides immediately upstream of the translation start codon ATG are numbered as "-1". The putative heat-responsive *cis*-elements are underlined. HSE (nGAAn core), heat shock element consisting of repeated core nGAAn in alternate orientation; HSE (PlantCARE), heat shock element found in PlantCARE database; CCAAT box (Place), CCAAT box found in PLACE database.

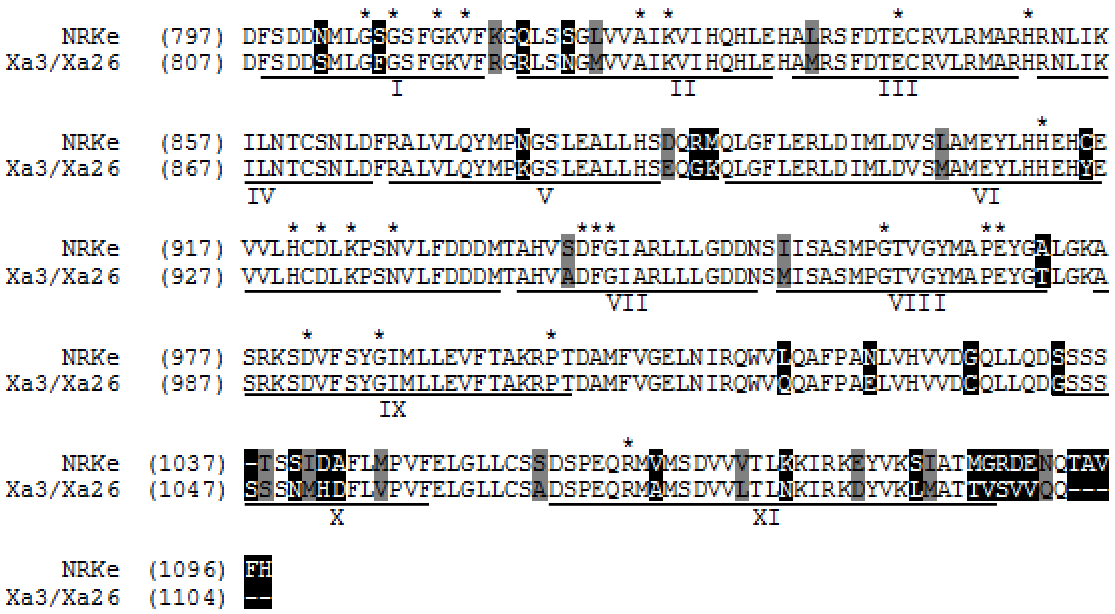

Figure S4: *Alignment of kinase domains of NRKe and Xa3/Xa26.* The solid black shade indicates different amino acid residues and the gray shade indicates residues with similarity. Asterisks (*) indicate conserved amino acid residues of protein kinase (Hanks SK et al. *Science* 1998, **241**:42-52). The conserved subdomains are numbered and underlined according to Cao et al. (Cao Y et al. *Theor Appl Genet* 2007, **115**:887-895)..

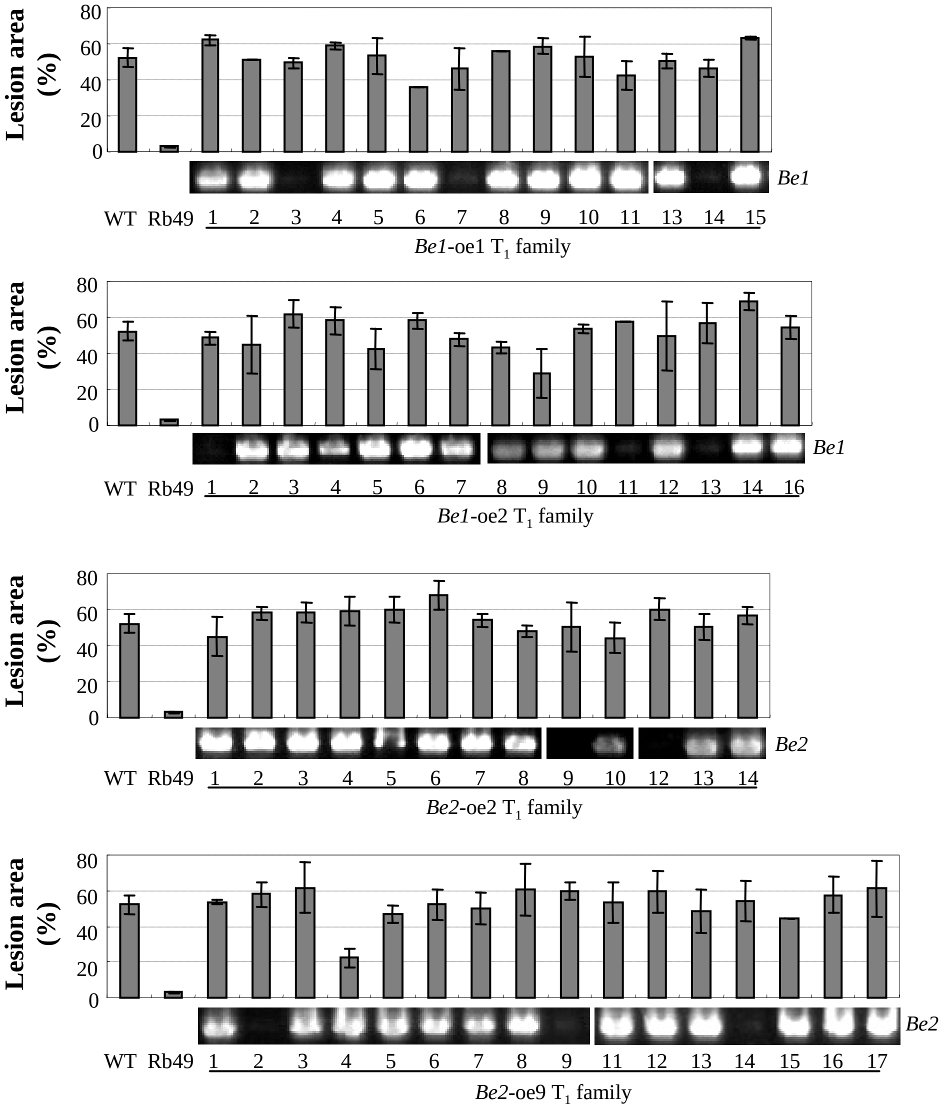

Figure S5: *Overexpression of Be1 or Be2 could not influence rice response to Xoo strain PXO61.* Positive transgenic plants were determined by PCR amplification of *Be1* or *Be2* using gene-specific primers (Additional file 1, Table S1). Wild type (WT) is Mudanjiang 8. Rb49 is a transgenic line carrying *Xa3/Xa26* driven by its native promoter in Mudanjiang 8 background.
